# Endoparasites in domestic cats (*Felis catus*) in the semi-arid region of Northeast Brazil

**DOI:** 10.1590/S1984-29612023065

**Published:** 2023-11-27

**Authors:** Welitânia Inácia Silva, Estefany Ferreira Lima, Jordania Oliveira Silva, Mariana de Melo Alves, Carla Lícia Pinheiro Alves, Ana Luzia Peixoto Silva, Jeizom Abrantes Lima, Thais Ferreira Feitosa, Vinícius Longo Ribeiro Vilela

**Affiliations:** 1 Programa de Pós-graduação em Ciência e Saúde Animal, Universidade Federal de Campina Grande – UFCG, Patos, PB, Brasil; 2 Departamento de Medicina Veterinária, Instituto Federal da Paraíba – IFPB, Sousa, PB, Brasil

**Keywords:** *Ancylostoma* spp., helminths, protozoa, one health, *Spirometra* spp., zoonosis, *Ancylostoma* spp., helmintos, protozoários, saúde única, *Spirometra* spp., zoonose

## Abstract

The aim of this study was to evaluate the prevalence of endoparasites in domestic cats (*Felis catus*) in the city of Sousa, state of Paraíba, Northeast Brazil. A total of 207 samples of fresh feces were randomly collected from domestic and semi-domiciled cats. The samples were analyzed by simple centrifugation and centrifuge-flotation in sucrose solution for the diagnosis of helminth eggs and enteric protozoan oocysts and cysts. Epidemiological information was obtained to determine risk factors related to infections. Among the samples collected, 81.6% (169/207; 95% CI: 77.4-83.8) were positive for at least one parasite genus. *Ancylostoma* spp. was the most prevalent, at 67.1% (139/207), followed by *Taenia* spp. at 28.5% (59/207), and *Spirometra* spp. and *Platynosomum sp.*, both at 17.3% (36/207). The variables associated with helminth infection were a historic lack of deworming (*Odds ratio* = 12.25) and the presence of dry fur (*Odds ratio* = 2.15). No risk factors were observed for enteric protozoa infection. This study demonstrated a high prevalence of endoparasites in domestic cats in the city of Sousa, state of Paraíba, and associated risk factors, thus establishing an overview of the main helminths and protozoa that affect cats in this region.

## Resumo

O objetivo deste estudo foi avaliar a prevalência de endoparasitos em felinos domésticos (*Felis catus*) na cidade de Sousa, estado da Paraíba, Nordeste do Brasil. Foram coletadas 207 amostras de fezes frescas de gatos domiciliados e semidomiciliados, de forma aleatória. Analisaram-se as amostras por centrifugação simples e centrifugo-flutuação, em solução de sacarose para o diagnóstico de ovos de helmintos e oocistos e cistos de protozoários entéricos. Informações epidemiológicas foram obtidas para a determinação dos fatores de risco relacionados às infecções. Dentre as amostras coletadas, 81,6% (169/207; IC 95%: 77,4-83,8) foram positivas para pelo menos um gênero de parasito, sendo o mais prevalente *Ancylostoma* spp., com 67,1% (139/207), seguido de *Taenia* ssp. 28,5% (59/207), *Spirometra* spp. e *Platynosomum* sp., ambos com 17,3% (36/207). As variáveis que apresentaram associação com a infecção por helmintos foram o não histórico de vermifugações (*Odds ratio* = 12,25) e a presença de pelos secos (*Odds ratio* = 2,15). Não foram observados fatores de risco para as infecções por protozoários entéricos. Este estudo demonstrou uma alta prevalência de endoparasitos em felinos domésticos, na cidade de Sousa, Paraíba, associados ao fatores de risco, estabelecendo-se assim, um panorama dos principais helmintos e protozoários que acometem gatos nesta região.

## Introduction

Brazil has around 149.6 million pets, of which 27.1 million are cats, with a growth rate of 6% in recent years (Instituto Pet Brasil, 2022). Felines play an important role in society, being part of the family environment (Abbas et al., 2022). This proximity may pose risks to human health, since cats are hosts of several zoonotic parasites, which in addition to directly affecting the health of these animals may cause variable disturbances, from subclinical infections to more severe gastrointestinal alterations, such as severe diarrhea, anemia, and compromised growth. The parasites can also lead to death, mainly in young animals (Sauda et al., 2019).

Cats are susceptible to several gastrointestinal parasites, including: *Ancylostoma* spp., *Toxocara* spp., *Dipylidium* spp., *Toxascaris* spp., *Strongyloides* spp., *Platynosomum sp*., *Spirometra* spp. and *Trichuris* spp., in addition to protozoa such as *Toxoplasma gondii*, *Cystoisospora* spp., *Cryptosporidium* spp. and *Giardia sp*. (Monteiro et al., 2016; Pereira et al., 2017; Silva et al., 2023a). Transmission can occur via the fecal-oral route, through the skin penetration of larvae, and by ingestion of intermediate and paratenic hosts (Arruda et al., 2021).

Some of these parasites can cause serious diseases in humans, as in the case of *T. gondii*, which can cause neurological and reproductive changes, and infects a third of the world's population (Lourido, 2019). Still within the protozoa, *Giardia sp.* and *Cryptosporidium* spp. also pose a risk to public health because they are responsible for cases of human parasitic diarrhea (Taghipour et al., 2021; Fusaro et al., 2022). The most prevalent helminths are *Ancylostoma* spp. and *Toxocara* spp. (Monteiro et al., 2016), which are acquired through direct contact with the infective forms of the parasites in the environment, and through ingestion of eggs containing the infective larvae in contaminated food and water. The latter are responsible for causing cutaneous larva *migrans* and visceral/ocular larva *migrans*, respectively (Mello et al., 2022).

Studies on the prevalence of endoparasites with an adequate sampling of domestic felines in Brazil are scarce, especially when compared to studies with dogs. This may be related, in part, to the greater difficulty in handling felines due to their temperament, which results in fewer parasitological exams (Dantas-Torres, 2020). There are no studies on the prevalence of enteric parasites that affect felines in the semi-arid region of Paraíba. In other regions of Northeast Brazil, the prevalence of entoparasites in cats ranged from 23% to 65.3% (Monteiro et al., 2016; Lima et al., 2017; Teófilo et al., 2017). Thus, this study aimed to describe the prevalence and risk factors associated with helminth and enteric protozoan infections in domestic cats (*Felis catus*) in the semi-arid region of the State of Paraiba, Northeast Brazil.

## Material and Methods

### Experiment location

The research was carried out in the municipality of Sousa, State of Paraiba, Northeast Brazil (6°45'50”S; 38°13'30”W), from April to October 2022. The analyses were carried out at the Laboratory of Veterinary Parasitology of the Federal Institute of Education, Science and Technology of Paraiba, Sousa Campus.

### Sampling

The calculation for simple random sampling was used to determine the minimum number of samples to be collected:


$$n=\frac{\frac{Z^{2}\times P\left(100-P\right)}{d^{2}}}{1+\left(\frac{Z^{2}\times P\left(100-P\right)}{d^{2}\times N}\right)}$$ (1)

*n =* sample size for finite population

*Z* = 1.96 (normal distribution value for the 95% confidence interval)

*P* = 50 (expected prevalence of 50% to achieve sample maximization)

*d* = 10 (maximum value of the 10% absolute error to estimate proportion)

*N* = population size

The population size considered the cat-human ratio of 18.98, described by Canatto et al. (2012), page 1518. Given that the estimated population of the city of Sousa is 69,723 people (IBGE, 2022), a population of 3,674 cats was estimated. Thus, the minimum number of animals needed to participate in the study was 93. However, 207 samples were collected.

### Sample collection

The collections were carried out in randomly domiciled cats without street access, and semi-domiciled cats with free street access, both with responsible tutors, without predilection for sex, age, or breed. In households with up to three cats, one of them was selected, and in those with more than three cats, three were collected.

The animals were immobilized according to Feitosa (2014), and fecal samples were obtained by rectum washing, using 5 mL of 1:1 saline solution (0.9% NaCl + mineral oil), using a urethral lubricated probe. The samples were stored in dry screw-top containers, identified accordingly, and refrigerated at temperatures of 2 to 8°C until the analyses, within 24 h.

### Diagnosis of enteroparasites

Two techniques were used to identify evolutionary forms of gastrointestinal parasites: the simple sedimentation method (Hoffman et al., 1934) and the CFSS (centrifugation-flotation in sucrose solution) (Ogassawara et al., 1986).

Samples of 5 grams of feces and 50 mL of water were used in the simple sedimentation technique. After that, each sample was macerated, sieved, and added to a sedimentation cup, where another 50 mL of water was added to complete the cup volume, and left to rest for 30 min. Soon after, the supernatant was discarded, and another 100 mL of water was added. The sample was then left for another 30 min of rest. One or two more washes were performed until the supernatant had a light hue. For the slide preparation, the supernatant was discarded, and a drop of sediment was extracted using a Pasteur pipette, with a drop of Lugol superimposed on the coverslip. At least three slides were read to increase the probability of identifying parasitic forms as described by Tibiriça et al. (2009) and Alvares et al. (2021).

The CFSS (centrifugation-flotation in sucrose solution) method was performed using 2 grams of feces for each 20 ml of hypersaturated sucrose solution at a density of 1.203 g/cm^3^. Each sample was macerated, sieved, placed in a Falcon-type centrifuge tube with a volume of 15 ml and centrifuged at 2,000 rpm for 10 min. After centrifugation, the sample was collected from the surface using a loop of Henle and deposited on the slide overlaid with a cover slip (Ogassawara et al., 1986). Three slides were taken from each sample.

To measure the evolutionary forms of the parasites, an optical microscope LAB-DM300 was used, equipped with photomicrograph software, which could obtain images with up to 3.2 million pixels. All photomicrographs were produced using 40× and 100× lenses (400× and 1,000× magnifications) and the measurements were made using the Mv Image R software tools, similar to what was described by Araújo et al. (2020), Melo et al. (2022) and Silva et al. (2022). The microscopic diagnoses of the evolutionary forms were established based on morphometric data (Urquhart et al., 1991; Fortes, 2004; Bowman & Georgi, 2009).

### Epidemiological questionnaire and physical examination

During the collections, a structured epidemiological questionnaire was administered to collect information about the variables that could act as possible risk factors and to obtain information about the animals in the study. The main variables investigated were food, including feed and other foods; contact with other cats or dogs; deworming of animals; environmental cleaning; vaccinations, including anti-rabies and others; castrations; and birth history. The information obtained was entered into an electronic form prepared in the Microsoft Access® program and used in the analysis of risk factors.

General clinical assessments were also carried out to obtain information about nutritional status, hair condition, presence of secretions, attitude, motility, mucosal color, heart rate, respiratory rate, rectal temperature, degree of hydration, and palpation of lymph nodes, in addition to other information such as the presence of ectoparasites at the time of the examination.

### Statistical analysis

The study of risk factors associated with infection by gastrointestinal parasites was carried out using data from epidemiological questionnaires, in two stages: univariate analysis and multivariate analysis. In the univariate analysis, each independent variable was crossed with the dependent variable (positivity), and those that presented a p-value ≤ 0.20 in the chi-square test or Fisher’s exact test (Zar, 1999) were selected for the multivariate analysis using multiple logistic regression (Hosmer & Lemeshow, 2000). This threshold for variable selection in the multivariate analysis through multiple logistic regression was chosen based on established statistical practices to ensure a broader consideration of potential predictors. This approach is in line with recommendations to include variables with p-values up to 0.20 to minimize the risk of excluding potentially important predictors in the initial stages of analysis. The final model was adjusted using the Hosmer and Lemeshow coefficient to better fit the value to p≥0.05.

The collinearity of the independent variables was determined by correlation analyses. When the correlation coefficient was < 0.9, one of the variables was eliminated according to biological plausibility (Dohoo et al., 1997). The significance level adopted in the multiple analysis was 5%. The results were analyzed using the GraphPad Prism® 9.0 program.

## Results

It was observed that, of the 207 fecal samples collected, 81.6% (169/207; CI: 77.4-83.8) were positive for one or more genera of parasites. The prevalence of enteric helminths was 81.6% (169/207; 95% CI: 77.4-83.8) and that of enteric protozoa was 13.04% (27/207; 95% CI: 8.4-17.6).

**Figure 1:**
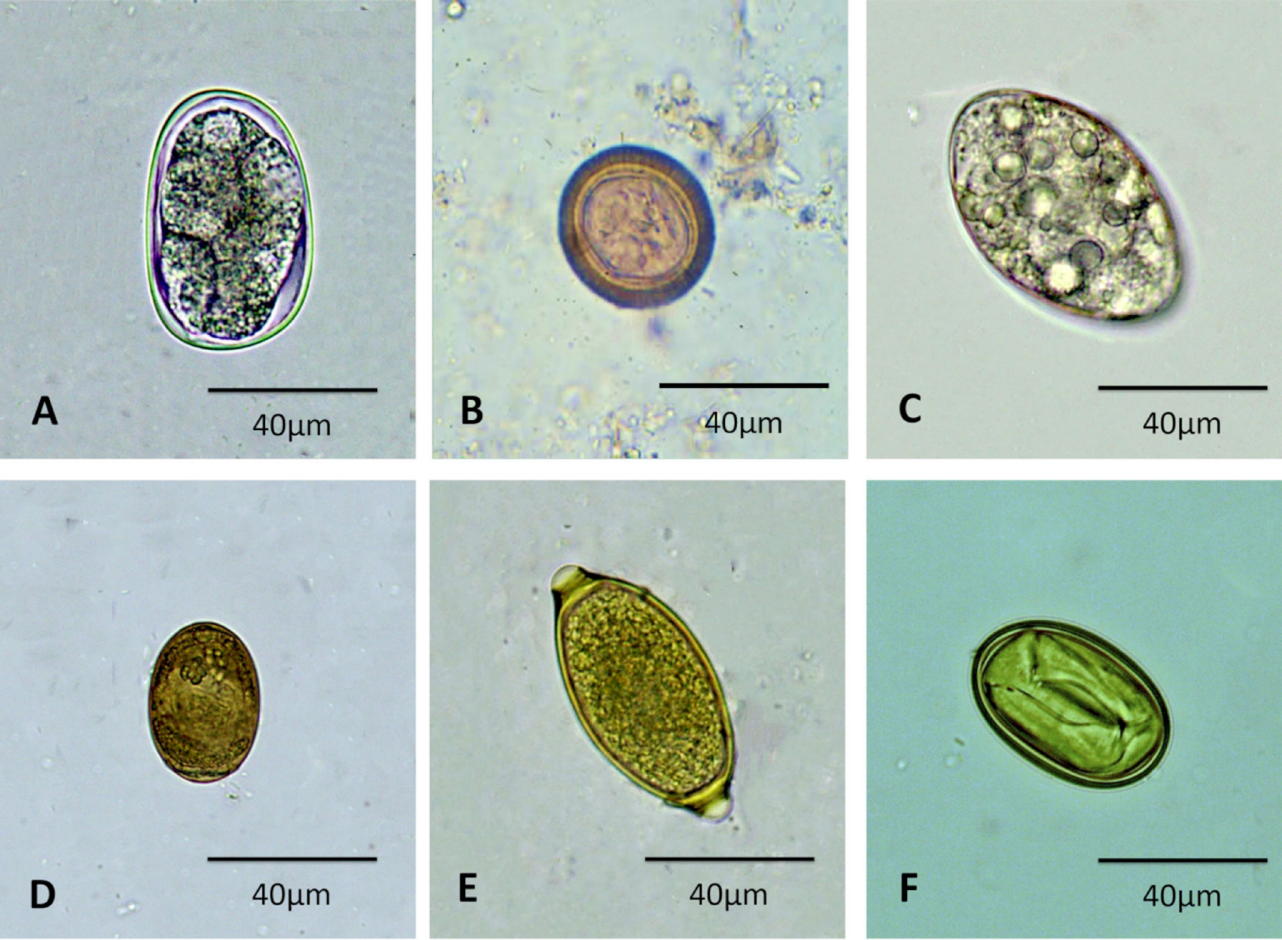
Helminth eggs diagnosed in fecal samples from domestic cats (*Felis catus*), in the city of Sousa, state of Paraíba, Northeast Brazil. (A) *Ancylostoma* spp.; (B) *Taenia* spp.; (C) *Spirometra* spp.; (D) *Platynosomum* sp.; (E) *Trichuris* spp.; (F) *Physaloptera* spp.

**Figure 2:**
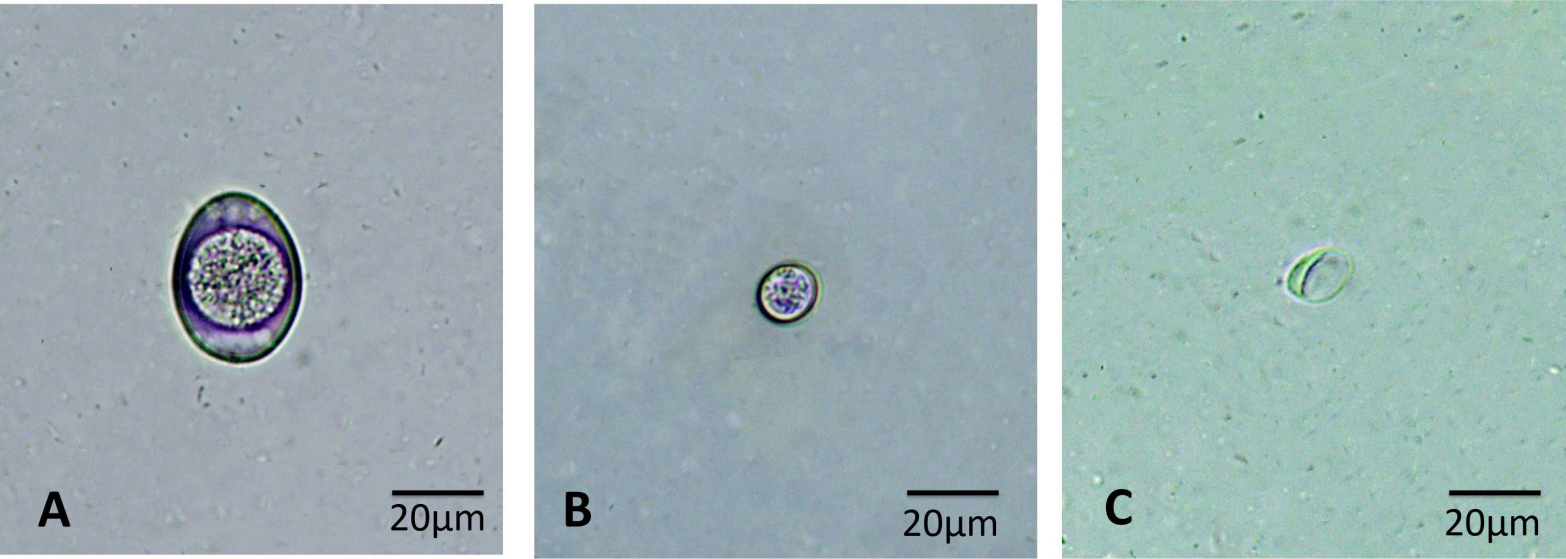
Oocysts and cysts of protozoa diagnosed in fecal samples from domestic cats (*Felis catus*) in the city of Sousa, state of Paraíba, Northeast Brazil. (A) non-sporulated oocyst of *Cystoisospora* spp; (B) unsporulated coccidial oocysts; (C) cyst of *Giardia* sp.

The most prevalent helminths were *Ancylostoma* spp. (Figure 1A), followed by Taenia spp. (Figure 1B), *Spirometra* spp. (Figure 1C), *Platynosomum sp.* (Figure 1D), *Trichuris* spp. (Figure 1E) and *Physaloptera* spp. (Figure 1F). The most prevalent protozoa were *Cystoisospora* spp. (Figure 2A), coccidial oocysts (Figure 2B) and *Giardia sp.* (Figure 2C). The prevalence rates of helminth and protozoan infections are detailed in Table 1.

**Table 1:** Prevalence of the genera of helminths and protozoa diagnosed in 207 domestic cats (*Felis catus*) in the semi-arid region of Paraiba, Northeast Brazil.

Genres of Parasites		Number of positives	Prevalence (%)	CI_95%_
Helminths eggs	*Ancylostoma* spp.	139	67.1	60.7-73.5
	*Taenia* spp.	59	28.5	22.4-34.6
	*Spirometra* spp.	36	17.3	12.2-22.4
	*Platynosomum* sp.	36	17.3	12.2-22.4
	*Physaloptera* spp.	12	5.7	2.6-8.9
	*Trichuris* spp.	8	3.8	1.2-6.4
Protozoa oocysts	*Cystoisospora* spp.	23	11.1	7.8-14.4
	coccidial oocysts	5	2.4	1.7-3.1
cysts	*Giardia* sp.	2	1.0	0.7-1.3

CI_95%_: confidence interval at 95% probability.

Of the positive samples, 49.8% (83/167; 95% CI: 45.7-53.9) showed co-infection with two or more parasites. The most prevalent co-infections were *Ancylostoma* spp. + *Taenia* spp., with 23% (39/169) positivity, followed by *Ancylostoma* spp.+ *Spirometra* spp., with 11.8% (20/169). Co-infections of helminths and protozoa were 10.7% (18/169) among *Ancylostoma* spp. and *Cystoisospora* spp.

The variables that showed significance for helminth and protozoan infections in the univariate analysis (p ≤ 0.20) are described in Table 2. Subsequently, they were included in the multivariate analysis (Table 3), where the risk factors associated with helminth infection obtained were no history of deworming and the presence of dry fur. As for infection by protozoa, no associated risk factors were observed.

**Table 2:** Univariate analysis of risk factors associated with helminth and protozoa infections in 207 domestic cats (*Felis catus*) in the city of Sousa, state of Paraiba, Northeast Brazil.

Variables/category	Total cats	Helminths		Protozoa	
		Positives (%)	*p*	Positives (%)	*p*
Food					
Commercial	87	75 (86.2)		9 (10.4)	
Homemade	10	9 (90.0)	0.108*	0 (0)	0.173*
Both	86	64 (76.1)		15 (17.5)	
Contact with felines					
Yes	180	150 (83.3)		26 (14.4)	
No	27	19 (70.3)	0.114*	1 (3.7)	0.215
Contact with dogs					
Yes	188	151 (80.3)		27 (14.3)	
No	19	16 (84.2)	0.769	0 (0)	0.082*
Previous Deworming					
Yes	177	139 (78.5)		25 (14.2)	
No	29	29 (100)	0.003*	2 (6.8)	0.383
Environmental cleaning					
Yes	156	125 (80.1)		24 (15.3)	
No	26	24 (92.3)	0.174*	2 (7.7)	0.536
Vaccination					
Anti-rabies	70	56 (80.0)		10 (14.2)	
Others	5	3 (60.0)	0.288	2 (40.0)	0.178*
Castration					
Yes	44	32 (72.8)		4 (9.1)	
No	152	128 (84.2)	0.119*	19 (12.5)	0.791
Farrowing					
Yes	29	19 (65.5)		4 (13.7)	
No	94	76 (80.9)	0.126*	9 (9.5)	0.502
Fur					
dry	41	37 (90.2)		8 (19.5)	
bright	164	130 (79.3)	0.121*	19 (11.6)	0.198*
Hydration					
hydrated	153	121 (79.0)		21 (17.4)	
Dehydrated	54	48 (89.0)	0.151*	06 (11.1)	>0.999

* Variables that presented *p* values ≤ 0.20 according to the chi-square test and/or Fisher's exact test.

**Table 3:** Multivariate analysis of risk factors associated with helminth infections in domestic cats (*Felis catus*) in the city of Sousa, state of Paraíba, Northeast Brazil.

Risk factors	Odds ratio	CI 95%	*p*
Helminths			
No deworming history	12.25	[8.55-15.95]	0.014
Presence of dry fur	2.15	[1.35-2.95]	0.047

CI_95%_: confidence interval at 95% probability.

## Discussion

There was a high prevalence (81.6%, or 169/207) of parasitized felines in the present study. In studies carried out in other Brazilian states, lower prevalence values were obtained. In Pernambuco, a prevalence of 65.1% (113/173) was obtained (Monteiro et al., 2016); in São Paulo, 18.7% (91/502) (Gennari et al., 2016); in Rio de Janeiro, 24.5% (51/208) (Arruda et al., 2021); and in Rio Grande do Sul, 31.8% (108/339) (Marques et al., 2017). The present study used two different diagnostic techniques, simple sedimentation and CFSS, and three slides were performed, which increased the sensitivity of the results and the high positivity.

The most prevalent parasite was *Ancylostoma* spp., with 67.1% positivity. Similar results were found by Monteiro et al. (2016) and Campos et al. (2016), with 67.2% and 54%, respectively. This high prevalence may be related to the monoxene life-cycle and the high egg’s laying by female, which increases environmental contamination (Bowman et al., 2002). This high prevalence creates a public health problem because it is a zoonosis that causes cutaneous larva *migran*s in humans, where infection can occur through direct contact with feces containing the infective form in the environment, such as in public squares, vacant lots and sandboxes, among other sites (Campos et al., 2016).

According to Campos et al. (2016), the most frequent parasites in cats are *Ancylostoma* spp. and *Toxocara* spp., which are both zoonotic. However, in this study, no animal was positive for *Toxocara* spp., a result that differed from those of several other studies that obtained high positivity rates (Campos et al., 2016; Monteiro et al., 2016; Pivoto et al., 2013). In the present study, most cats (87.4%; 181/207) were over 6 months old, which is one of the factors that can lead to low rates or the absence of positivity. According to Pereira et al. (2017), age influences the load of eggs eliminated, as when animals become adults, the larvae tend to encyst in the tissues, thus reducing the rate of evolution to adult parasites in the small intestine. In addition, the hot semi-arid climate of the region, with a rainy season from January to May, when occurs an average of 98.6% of annual rainfall (mean of 870 mm), and a dry season from June to December, with mean temperature of 27 ºC (INMET, 2022), may not be suitable specifically for *Toxocara* spp. eggs, possibly resulting in structural instability, metabolic deactivation, and desiccation, hindering their dissemination and leading to the absence of the parasite.

The most common cestode found was *Taenia* spp., at 28.5%. This was a high prevalence compared to that found in other studies in Brazil. Campos et al. (2016) found 16%, while Stalliviere et al. (2009) obtained only 0.7%. Similar prevalence was observed in the United States, at 36% positivity, and in Belgium, at 28% (Vanparijs et al., 1991; Loftin et al., 2019). One of the factors that may have contributed to the high prevalence is the hunting behaviour, reported in 60.8% (126/207) of the animals, and thus, the potential for ingestion of intermediate hosts. According to Hoopes et al. (2015), cats that engage in hunting habits may be consuming rodents that serve as intermediate hosts for *Taenia* spp., potentially acquiring infections through this route.

*Spirometra* spp. was one of the most frequent parasites in this study, with 17.3% positivity. This was considered a high prevalence compared to those found in other studies in Brazil, in which rates ranged from 1.4 to 4.8% positivity (Ramos et al., 2013; Ferraz et al., 2021). In a systematic global study, it was observed that cats had a higher rate of infection by this parasite than dogs (Badri et al., 2022) and that these animals play an important role in the contamination of the environment. It is worth mentioning *Spirometra* spp.’s zoonotic potential. It is what causes the disease Sparganosis in humans, which can be acquired through the ingestion of undercooked meat and infected water containing copepods. There is no treatment other than surgically removing the larvae (Mentz et al., 2011).

Another important parasite that affects the health of felines is *Platynosomum sp*., which was found to have a pooled global infection prevalence in domestic felines (*F. catus*) of 17.8% (Silva et al., 2023b), very similar to the rate obtained in this study, which was 17.3%. Studies by Lima et al. (2021b) and Lins et al. (2021) in Mato Grosso do Sul obtained higher values of 26.9% and 20.9% respectively, while in Ceará, a cross-sectional study detected 42.6%, but the form of diagnosis was necropsy (Braga et al., 2016). The difference between the frequencies must have been due to the method used for diagnosis and the intermittent elimination of eggs (Lima et al., 2021a). It is necessary to emphasize the importance of infection by this parasite in the health of cats, because clinical changes can range from asymptomatic to severe liver changes (Sobral et al., 2019). These results indicate veterinarians should include this parasitosis as a differential diagnosis and perform preventive diagnoses and proper deworming.

*Cystoisospora* spp. was the most prevalent protozoan with 11.1% of positive selections. A superior result was found by Gennari et al. (1999), who determined that rate to be 38.5%. Other studies carried out by Gennari et al. (2016) and Arruda et al. (2021) obtained prevalence rates of 4.8% and 4.6%, respectively. In other countries such as Egypt, the United States, Italy, and Greece, 9.1%, 16.5%, 10.2% and 16.4% positivity rates, respectively, were found (Symeonidou et al., 2018; Hoggard et al., 2019; Genchi et al., 2021; Abbas et al., 2022). *Cystoisospora* spp. is a parasite of importance to feline health because the infection is usually asymptomatic, but in immunocompromised animals it can lead to the destruction of the intestinal epithelium, causing mild to severe diarrhea, vomiting and anorexia (Symeonidou et al., 2018).

Coccidial oocysts (10-12μm) were observed in 5 (2.4%) samples. At least three genera of coccidia with similar dimensions to those found in this study have been identified, which can be shed by cats: *Toxoplasma* sp., *Besnoitia* spp., and *Hammondia* spp. In the semi-arid region of the State of Paraíba, there was a high prevalence of anti-*T. gondii* antibodies in cats, with many animals showing high antibody titers, suggesting acute infections (Feitosa et al., 2014; Feitosa et al., 2021). However, due to the similarity between the genera, it was not possible to identify them solely based on morphological characteristics associated with the serology results in the studied region. According to Dubey & Sreekumar (2003), there is no easy way to distinguish *Hammondia* sp. from *Toxoplasma* sp. oocysts in cat feces except through bioassays in mice.

One of the risk factors associated with helminth infection in the multivariate analysis was the lack of deworming history. That is, 12.5% of cats that were not dewormed had more chances of becoming infected. According to studies carried out by Beugnet et al. (2014), cats that did not receive any anthelmintic treatment had a significantly greater risk of becoming infected with helminths. In the present study, most of the animals 14% (29/207) had never undergone anti-parasitic, and all of these animals tested positive for helminth eggs, which may have contributed to the high prevalence. For Strube et al. (2019), one of the reasons for the low adherence to deworming of animals by some tutors may be the lack of information on zoonotic risks and the potential impact on feline health.

Another factor associated with helminth infection was the presence of dry fur. Cats with this characteristic have 2.15% higher chances of being infected with helminths. The presence of dry fur can indicate a suboptimal or nutritionally inadequate health status, rendering the cat more susceptible to helminth infections due to a weakened immune system (Vojtkovská et al., 2020). It may also signify that the animals are struggling to groom themselves adequately, potentially increasing the risk of coming into contact with an environment contaminated with helminth eggs, resulting in a greater likelihood of infection.

## Conclusion

There was a high prevalence of intestinal parasites in domestic cats in the city of Sousa, state of Paraíba, Northeast Brazil. This study also demonstrated the prevalence of zoonotic parasites such as *Ancylostoma* spp. and *Spirometra* spp., and served as a warning for of need to adopt adequate prophylaxis and control measures. Cats with no prior deworming history and those displaying dry fur exhibit a higher positivity rate for helminth infections.
